# A comparison of stability metrics based on inverted pendulum models for assessment of ramp walking

**DOI:** 10.1371/journal.pone.0206875

**Published:** 2018-11-05

**Authors:** Nathaniel T. Pickle, Jason M. Wilken, Nicholas P. Fey, Anne K. Silverman

**Affiliations:** 1 Department of Bioengineering, The University of Texas at Dallas, Richardson, Texas, United States of America; 2 Department of Mechanical Engineering, Colorado School of Mines, Golden, Colorado, United States of America; 3 Department of Rehabilitation Medicine, Brooke Army Medical Center, San Antonio, Texas, United States of America; 4 Department of Mechanical Engineering, The University of Texas at Dallas, Richardson, Texas, United States of America; 5 Department of Physical Medicine and Rehabilitation, The University of Texas Southwestern Medical Center, Dallas, Texas, United States of America; University of Illinois at Urbana-Champaign, UNITED STATES

## Abstract

Maintaining balance on ramps is important for mobility. However, balance is commonly assessed using inverted pendulum-based metrics (e.g., margin of stability), which may not be appropriate for assessment of human walking on non-level surfaces. To investigate this, we analyzed stability on ramps using four different inverted pendulum models: extrapolated center of mass (XCOM), foot placement estimate (FPE), foot placement estimate neglecting angular momentum (FPE_NoH_), and capture point (CAP). We analyzed experimental data from 10 able-bodied individuals walking on a ramp at 0°, ±5°, and ±10°. Contrary to our hypothesis that the magnitude of differences between metrics would be greatest at ±10°, we observed the greatest magnitude of differences between metrics at 0°. In general, the stability metrics were bounded by FPE and CAP at each slope, consistent with prior studies of level walking. Our results also suggest that clinical providers and researchers should be aware that assessments that neglect angular momentum (e.g., margin of stability, XCOM) may underestimate stability in the sagittal-plane in comparison to analyses which incorporate angular momentum (e.g., FPE). Except for FPE_NoH_-CAP (r = 0.82), differences between metrics were only moderately correlated (|r|≤0.65) with violations of leg length assumptions in the underlying inverted pendulum models. The differences in FPE_NoH_ relative to FPE and CAP were strongly correlated with body center of mass vertical velocity (max |r| = 0.92), suggesting that model representations of center of mass motion influence stability metrics. However, there was not a clear overall relationship between model inputs and differences in stability metrics. Future sensitivity analyses may provide additional insight into model characteristics that influence stability metrics.

## Introduction

Walking on sloped surfaces, such as ramps, can be challenging due to the need to effectively raise or lower the body center of mass (COM) while maintaining balance. The difficulty of sloped walking is evident in biomechanical outcomes such as increased metabolic energy expenditure on inclines relative to level ground [1] and increased power absorption at the knee joint on declines [2]. While various aspects of ramp walking have been studied extensively [1–5], less is known about how sagittal-plane stability is maintained during this challenging task. One reason for the lack of knowledge regarding stability on non-level surfaces may be that a commonly used method for assessing stability, the margin of stability [6], is formulated using a passive inverted pendulum-based model of the human body that may not generalize to non-level walking.

The inverted pendulum is a mathematically simple system and the conditions necessary for its mathematical stability can be proven, in some cases analytically. The margin of stability is based on the extrapolated center of mass (XCOM), which was originally derived for standing balance. The model used in this calculation assumes that the force of gravity acting on the body center of mass (COM) and the vertical ground reaction force (GRF) are the only forces that generate moments about the ankle joint, which is taken to be the pivot point of a rigid inverted pendulum [6]. In the static case the person is presumed to be stable as long as the vertical projection of the COM onto the ground is within the boundaries of the stance foot, and the distance from the COM projection to the foot boundary is referred to as the margin of stability. To account for a nonzero COM velocity during dynamic tasks, the XCOM is used instead of the COM projection. The XCOM location is given by

$$XCOM\text{=}x\text{+}\frac{V_{x}}{\omega_{0}}$$ (1)

where *x* and *v*_*x*_ are the horizontal location and velocity, respectively, of the COM, and the natural frequency *ω*_0_ of the inverted pendulum is given by

$$\omega_{0}=\sqrt{\frac{g}{L}}$$ (2)

where *g* is the acceleration due to gravity and *L* is a constant leg length value.

The XCOM is commonly used to investigate stability in both the sagittal [7–10] and frontal [11–13] planes during movement. XCOM is popular in experimental studies because the calculation requires only kinematic quantities including the position and velocity of the COM, locations of the foot boundary, and a constant leg length value calculated from a static trial. XCOM also relates quantities that can potentially be targeted during training, namely COM velocity and foot placement. The passive model underlying XCOM calculations relies on assumptions that likely limit its applicability to non-level walking in humans. For example, although the COM behaves similarly to an inverted pendulum during the single leg stance phase of level-ground walking, there is still a change in leg length over the stance phase due to lower-limb joint flexion [14]. This assumption is further violated when walking up an incline as the lower-limb joints become more flexed [4]. Furthermore, key characteristics of sloped walking such as net knee joint power absorption on declines [2] and hip extensor power on inclines [3] cause the human body to behave less like a passive inverted pendulum on these surfaces.

Other stability metrics have been developed based on different formulations of the inverted pendulum model that may be better suited to analyzing human walking on non-level surfaces. For example, the model used to calculate the foot placement estimate (FPE) gives the location on the ground where a rigid leg should be placed so that all kinetic energy is converted into potential energy and the body comes to a rest directly above the FPE location [15]. Thus, the FPE provides a measure of how easily a person will be able to stop during walking. The FPE location is strongly correlated with human foot placement in the sagittal plane during a variety of walking tasks [16]. Although the leg in the model used for FPE is rigid, the effective leg length is computed from the instantaneous height of the COM, unlike the constant leg length (computed from a static posture) used to compute XCOM. The model used to calculate FPE also incorporates rotational momentum of the body rather than treating the body as a point mass. The inclusion of rotational momentum and the ability to vary leg length may provide benefits for assessing sloped walking when compared to XCOM.

A third inverted pendulum-based stability metric is the capture point (CAP). Similar to the FPE, the CAP is the location where a biped should step in order to bring the body to rest [17]. However, rather than assuming a rigid leg, the CAP is formulated using the linear inverted pendulum model [18]. The linear inverted pendulum is an abstraction of the traditional inverted pendulum model in which the length of the rod (leg) is allowed to vary while a quantity called “orbital energy” is conserved [19]. The capture point algorithm proposed by [17] achieves a closed form solution by assuming zero vertical displacement of the COM, but the algorithm can be extended to arbitrary COM trajectories and terrain by numerically solving the equations of motion [20]. This numerical solution allows use of a model that is more general than those used in XCOM or FPE due to the fact that leg length is not fixed, but depends only on the location of the COM relative to the foot. However, the model used to calculate CAP does not incorporate rotational momentum, unlike the model used in the FPE.

Because of the differences in their underlying model formulations, stability metrics computed using the XCOM, FPE, and CAP models may provide different results when assessing stability during ramp walking. In order to characterize the relationships between these different metrics during ramp walking, we performed three different analyses. First, we tested for significant differences between the metrics at each ramp angle. We hypothesized that there would be no differences between metrics on level ground, but significant differences between metrics on inclines and declines. Second, we evaluated the relative differences between metrics by comparing both the signed and absolute differences between models. We hypothesized that there would be greater differences between metrics at the largest slopes (±10°) where the assumptions of the underlying models are violated to a greater extent. Finally, in order to help explain the differences between metrics, we tested for correlations between the metric differences and various inputs to the underlying models across all slopes, to see which quantities are most strongly linked to differences between metrics regardless of slope. In particular, we hypothesized that there would be changes in effective leg length at heel strike to have a significant correlation with differences between metrics (i.e., greater violations of leg length assumptions would lead to greater differences between metrics).

## Methods

The experimental protocol was completed by able-bodied individuals (2 female/8 male, mean±SD age 24±5 years, height 1.80±0.09 m, body mass 91±10 kg). The protocol was approved by the Institutional Review Board at Brooke Army Medical Center and all participants provided their informed consent. Participants were excluded based on known history of balance or visual impairment, neurological disorders, chronic musculoskeletal conditions, cardiac or pulmonary conditions.

Participants performed four walking trials at each slope of 0˚, ±5˚, and ±10˚ on a 4.88 m (16 ft) ramp while we recorded whole-body kinematics at a frequency of 120 Hz (Motion Analysis Corp., Santa Rosa, CA). The order of non-level conditions was randomized between participants. An auditory cue was used to control horizontal velocity at a speed computed using a Froude number of 0.16 on level and slopes [21], resulting in a mean±SD horizontal velocity of 1.21±0.08 m/s. Fifty-seven reflective markers were used to define and track 13 body segments [22,23]. Kinematic marker trajectories were filtered using a 4th-order low pass Butterworth filter with a 6 Hz cutoff frequency. Multi-segment models of each subject were created in Visual3D (C-Motion, Inc., Germantown, MD), with segment masses determined as a percentage of total body mass [24] and rotational inertia properties computed based on modeled segment geometry.

### Extrapolated center of mass (XCOM)

**Fig 1:**
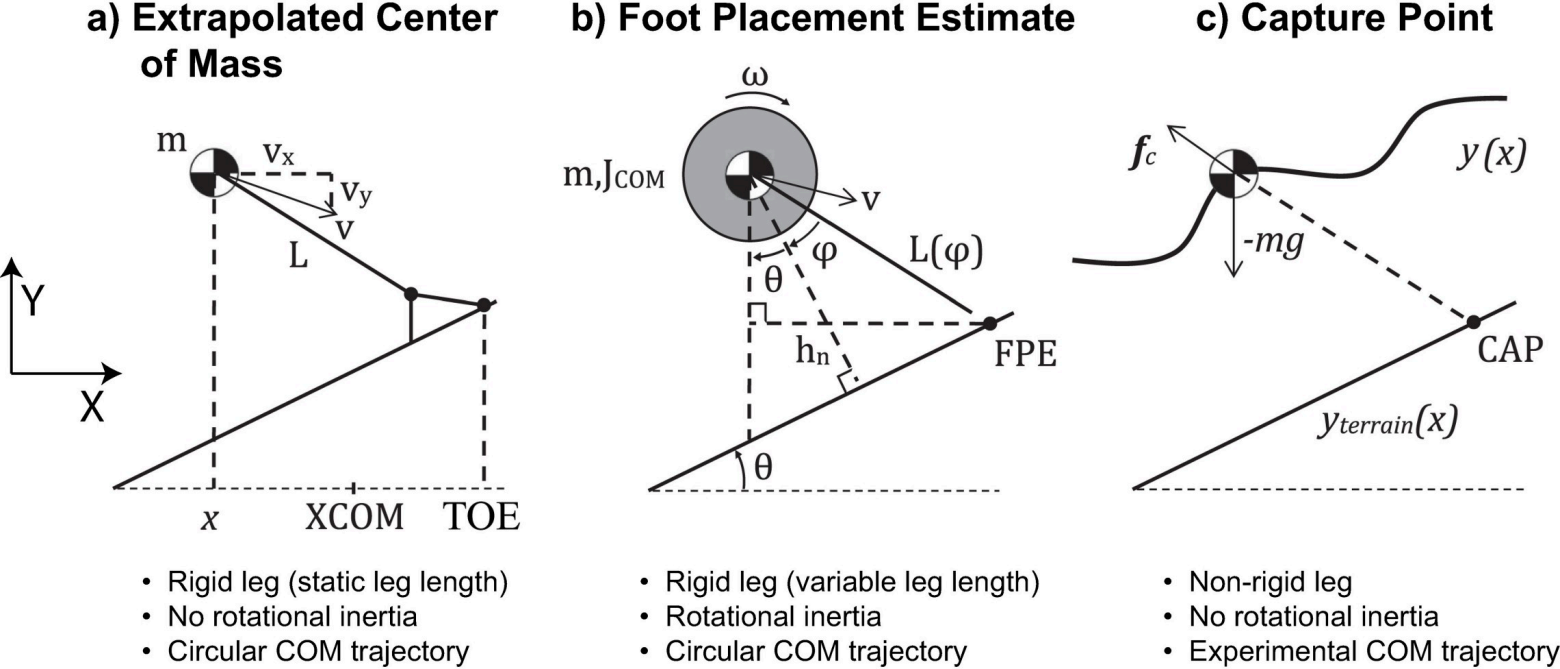
Inverted pendulum model diagrams. Diagrams illustrating the models used for calculating stability metrics: the extrapolated center of mass (XCOM, a), foot placement estimate (FPE, b), and capture point (CAP, c). The ramp angle and the angle of the leg relative to the vertical have been exaggerated for clarity. A summary of key model characteristics is given below each diagram.

The inverted pendulum-based stability metric calculations were performed in MATLAB (The Mathworks, Inc., Natick, MA). The XCOM location in the x-direction (Fig 1A) was calculated using Eqs (1) and (2) (see Introduction), with the leg length constant *L* calculated as the mean sagittal-plane distance from the lateral malleolus markers to the body COM during a static trial based on [3].

### Foot placement estimator (FPE)

The leg length *L(φ)* of the model used to calculate FPE (Fig 1B) was calculated as

$$\text{L(}\varphi \text{)=}\frac{h_{n}}{\cos\varphi }$$ (3)

where *φ* is the angle between the leg and a line through the body COM that is perpendicular to the ramp. The perpendicular distance *h*_*n*_ of the COM from the ramp is given by

$${\text{h}}_{\text{n}}\text{=cos(}\theta {\text{)[y-y}}_{terrain}\text{(}x\text{)]}$$ (4)

where *θ* is the ramp angle, *y* is the global *y*-coordinate of the COM and *y*_*terrain*_*(x)* is the global *y*-coordinate of the ramp (that is, the height of the ramp) as a function of the current global *x*-coordinate of the COM. The model used to calculate FPE assumes that angular momentum about the FPE location is conserved during foot contact,

$$H_{FPE1}=H_{FPE2},$$ (5)

where *H*_*FPE1*_ and *H*_*FPE2*_ are the angular momentum before and after contact, respectively. The equations derived in [16] describing foot contact were modified to allow for variable ramp angle *θ*:

$$mL\left(\varphi \right)[v_{x}\cos\left(\varphi +\theta \right)+v_{y}\sin(\varphi +\theta )]+J_{COM}\omega_{1}=\left(mL{\left(\varphi \right)}^{2}+J_{COM}\right)\omega_{2}.$$ (6)

In Eq (6), *m* is the total body mass, and *v*_*x*_ and *v*_*y*_ are, respectively, the global horizontal and vertical velocities of the COM. The term $J_{COM}\omega_{1}$ is the sagittal-plane whole-body angular momentum about the COM, where *J*_*COM*_ is the net rotational inertia of the system of rigid bodies in the sagittal plane and *ω*_*1*_ is the moment of inertia-weighted average angular velocity [16,25]. Rearranging Eq (6) gives an expression for *ω*_*2*_, the angular velocity of the model after foot contact. Applying conservation of energy after foot contact gives

$$T_{2}+V_{2}=mgh_{peak},$$ (7)

where *T*_*2*_ and *V*_*2*_ are the kinetic and potential energy, respectively, of the model after foot contact, and *h*_*peak*_ is the maximum height of the COM above the ramp and is equal to *L(φ)*. Thus, Eq (7) can be re-written as

$$\frac{1}{2}(J_{COM}+mL\left(\varphi {)}^{2}\right)\omega_{2}^{2}+mgL\left(\varphi \right)\cos\left(\varphi +\theta \right)=mgL\left(\varphi \right)$$ (8)

and can be solved numerically for *φ*. The horizontal FPE location in global coordinates is given by

FPE=x+L(φ)sin(φ+θ) (9)

where *x* is the global *x-*coordinate of the COM at the instant of heel strike. We also performed this calculation with no angular momentum term, i.e., setting J_COM_ = 0 in Eqs (6) and (8). The result is denoted FPE_NoH_.

### Capture point (CAP)

The generalized capture point algorithm derived in [20] was used to calculate the location of the capture point (Fig 1C). The COM dynamics in the horizontal direction are given by

$$a_{x}=\frac{\left(x-x_{CAP}\right)\left(g+\frac{d^{2}y}{dx^{2}}v_{x}^{2}\right)}{\left(y-y_{CAP}\right)-\left(x-x_{CAP}\right)\frac{dy}{dx}}$$ (10)

In Eq (10), *a*_*x*_ is the global *x-*acceleration of the COM, *x* and *y* are the global *x-* and *y-*coordinates, respectively, of the COM, *v*_*x*_ is the global *x*-velocity of the COM, and *x*_*CAP*_ and *y*_*CAP*_ are the global *x-* and *y-*coordinates, respectively, of the CAP on the ramp. An 8^th^-order Fourier series was fit to the experimental COM trajectory and used to describe the COM trajectory as a function of *x*, *y(x)*. Eq (10) was numerically integrated using the ode45 solver in MATLAB, with position and velocity of the COM at the instant of heel strike as initial conditions, to find the value for *x*_*CAP*_ that caused the COM to come to rest at *y(x*_*CAP*_*)*. The solution was considered to have converged if $\left|x-x_{CAP}\right|<0.01m$ and |*v*_*x*_| <0.01m/s as time went to infinity in the numerical integration.

### Analysis

We first computed the global *x*-coordinates (horizontal position in the laboratory frame of reference) of the XCOM, FPE, FPE_NoH_ and CAP at the instant of heel strike. These locations were then subtracted from the global *x*-location of the toe marker on the leading foot (TOE, Fig 1A) and normalized by static leg length to obtain *stability metrics*. The mean±SD static leg length was 0.97±0.05 m. The instant of heel strike was determined using automatic gait event detection in Visual3D (C-Motion, Inc., Germantown, MD) and verified visually. We elected to analyze the instant of heel strike because it allowed for direct comparison between the participant’s toe placement and the XCOM, FPE, FPE_NoH_ and CAP locations, where the inverted pendulum models guarantee the ability to stop during that step. Thus, larger positive stability metric values (toe anterior to XCOM, FPE, FPE_NoH_ or CAP location) indicated a “safer” or more conservative step, while more negative values (toe posterior to XCOM, FPE, FPE_NoH_ or CAP location) indicated a less conservative step.

We analyzed one left and one right heel strike from each of the four walking trials (8 total heel strikes) at each slope. The heel strikes were extracted from the middle portion of the ramp, when the participants were in contact with force plates embedded in the ramp[26] (force data are not presented here).For each trial, stability metrics were computed for the XCOM, FPE, FPE_NoH_, and CAP, and the difference between metrics for that trial was computed. We then averaged the differences across all 8 trials. To determine whether the metrics were significantly different at each slope, we performed one-sample *t*-tests to determine if the differences were significantly different from zero. We tested 6 differences (XCOM-FPE, XCOM-FPE_NoH_, XCOM-CAP, FPE-FPE_NoH_, FPE-CAP, FPE_NoH_-CAP) at each of the 7 walking conditions, and thus we used a Bonferroni correction for a total of 42 tests, resulting in α = 0.012. To evaluate which walking condition yielded the greatest magnitude of differences between metrics, we performed 2-way ANOVAs with main effects of difference and walking condition (α = 0.05) to compare the signed and absolute differences between models. When a significant main or interaction effect was found, pairwise *post hoc* comparisons were performed using Tukey’s HSD. Lastly, correlation analyses were performed to determine if the differences in metrics were correlated with various model inputs at heel strike: effective leg length (distance from COM to ankle joint center), horizontal and vertical COM velocity, and horizontal distance between toe and body COM. We considered r≥0.8 strong, 0.5≤r<0.8 moderate, and r<0.5 weak.

## Results

**Fig 2:**
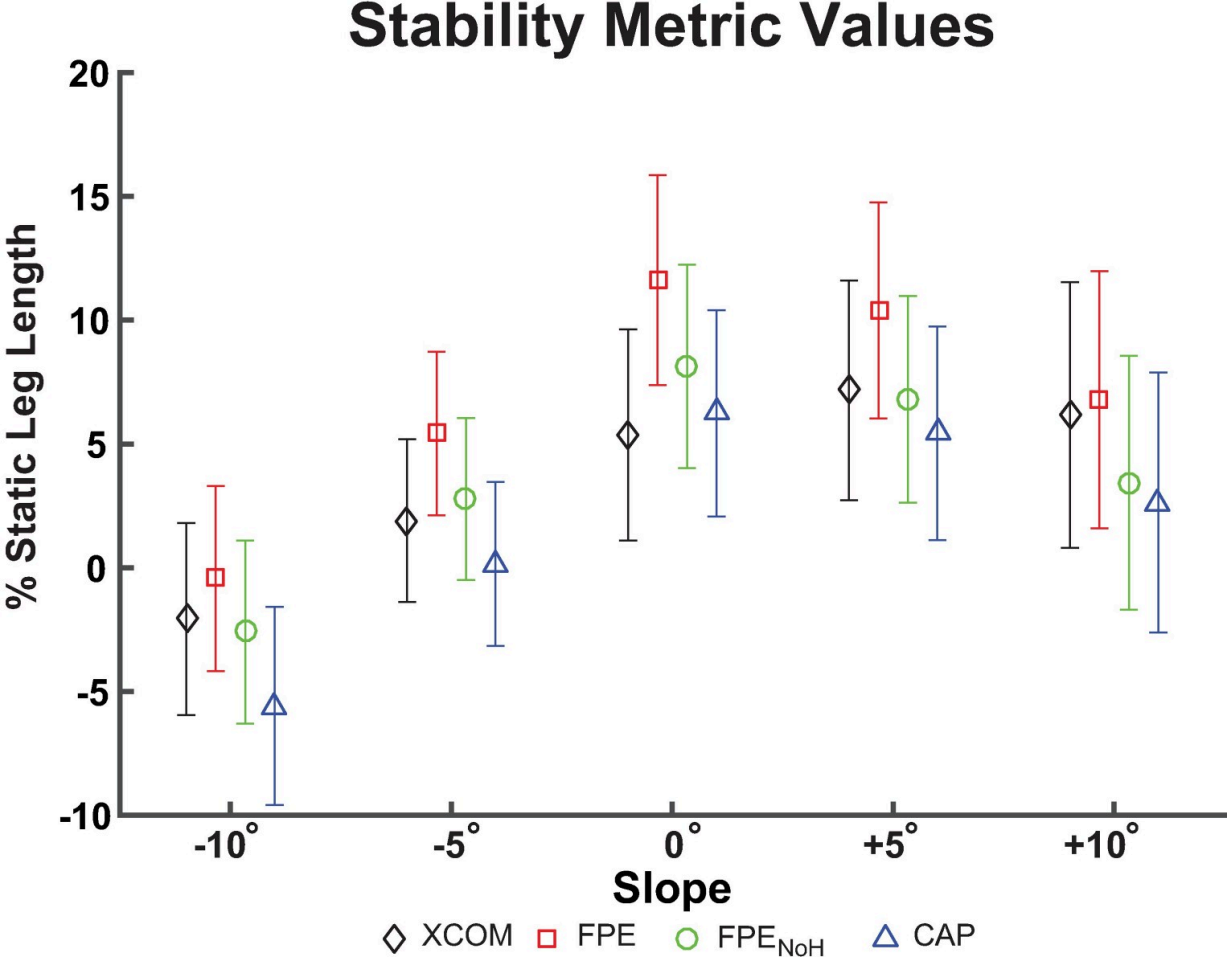
Stability metric values. Mean (±SD) stability metrics, computed as the distance between the toe marker and extrapolated center of mass (XCOM, black diamonds), foot placement estimate (FPE, red squares), foot placement estimate with no angular momentum (FPE_NoH_, green circles) and capture point (CAP, blue triangles) at heel strike, normalized to percent static (i.e., standing) leg length. Positive values are defined as “stable” (toe anterior to stability location), and negative values are “unstable” (toe posterior to stability location) by the metric definitions.

The mean stability metric values were generally positive, except for -10° (Fig 2), indicating a “stable” configuration at heel strike on most ramp angles.

### Differences between metrics at each slope

One-sample *t*-tests of the differences between metrics showed that all differences were significantly different from zero except for XCOM-FPE_NoH_ at -10° and +5°, and XCOM-FPE at +10° (Table 1).

**Table 1:** *p*-values from one-sample *t*-tests of differences between stability metrics.

	-10°	-5°	0°	+5°	+10°
XCOM—FPE	**0.000**	**0.000**	**0.000**	**0.000**	0.026
XCOM—FPE_NoH_	0.039	**0.001**	**0.000**	0.153	**0.000**
XCOM—CAP	**0.000**	**0.000**	**0.000**	**0.000**	**0.000**
FPE—FPE_NoH_	**0.000**	**0.000**	**0.000**	**0.000**	**0.000**
FPE—CAP	**0.000**	**0.000**	**0.000**	**0.000**	**0.000**
FPE_NoH_—CAP	**0.000**	**0.000**	**0.000**	**0.000**	**0.000**

XCOM, extrapolated center of mass; FPE, foot placement estimate; FPE_NoH_, foot placement estimate with no angular momentum term; CAP, capture point

Values less than 0.001 reported as 0.000.

Bold values indicate a significant difference after Bonferroni adjustment.

**Fig 3:**
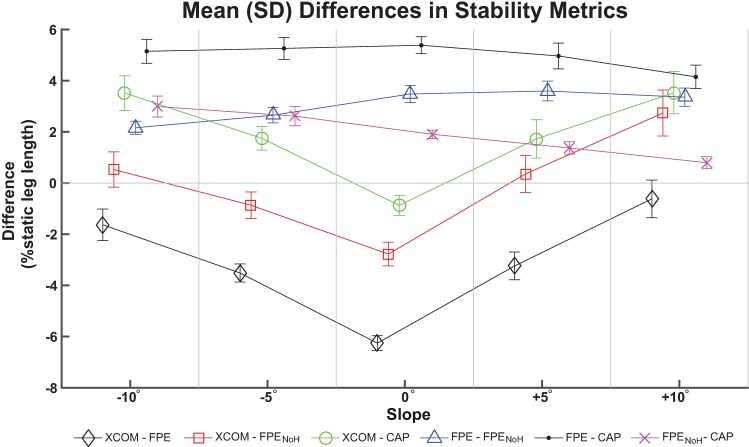
Mean (±SD) differences between stability metrics. Mean (±SD) signed differences between each pair of stability metrics at each of the slope angles investigated. Comparisons were made between extrapolated center of mass (XCOM), foot placement estimate (FPE), foot placement estimate with no angular momentum (FPE_NoH_) and capture point (CAP). Differences were computed by subtracting one metric from the other, e.g., XCOM-FPE denotes that the FPE metric was subtracted from the XCOM metric. Each metric was normalized to percent static (i.e., standing) leg length. Lines connecting data points are solely intended as a visual aid.

For the ANOVA comparing signed differences (Fig 3), there were significant main effects of metric and slope, as well as a significant interaction effect (all p<0.001). For each slope, the values of XCOM-FPE were significantly more negative (p<0.001) compared to all other differences at that same slope (Fig 3). Conversely, the FPE-CAP values were more positive compared to all other differences at each slope (all p<0.001) except +10°. At-10°, -5°, and 0°, the XCOM-CAP values were more positive compared to XCOM-FPE_NoH_ (p<0.001).

### Magnitude of differences between metrics

**Fig 4:**
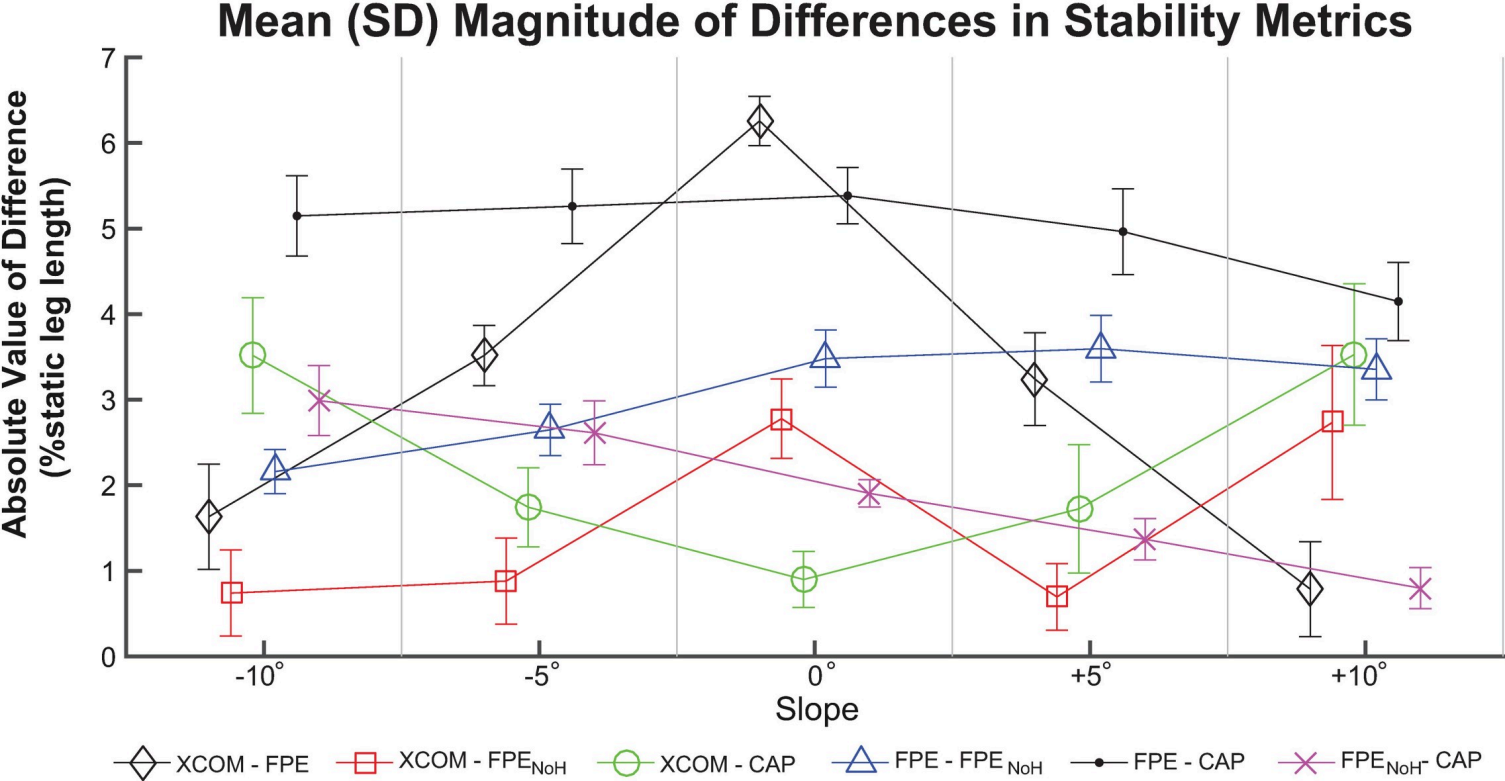
Mean (±SD) magnitude of differences between stability metrics. Mean (±SD) magnitude (i.e., absolute value) of differences between each pair of stability metrics at each of the slope angles investigated. Comparisons were made between extrapolated center of mass (XCOM), foot placement estimate (FPE), foot placement estimate with no angular momentum (FPE_NoH_) and capture point (CAP). Each metric was normalized to percent static (i.e., standing) leg length. Lines connecting data points are solely intended as a visual aid.

For the ANOVA comparing the magnitude of differences (Fig 4), the main and interaction effects were also all significant (p<0.001). The greatest magnitude of differences occurred in XCOM-FPE at 0° (p≤0.015 compared to all other differences). FPE-CAP was significantly larger than all other differences at -10°, -5°, and +5° (all p<0.001 within each slope), and was significantly larger compared to all differences except XCOM-CAP at 0° (p<0.001).

### Correlation between model inputs and stability metric differences

**Fig 5:**
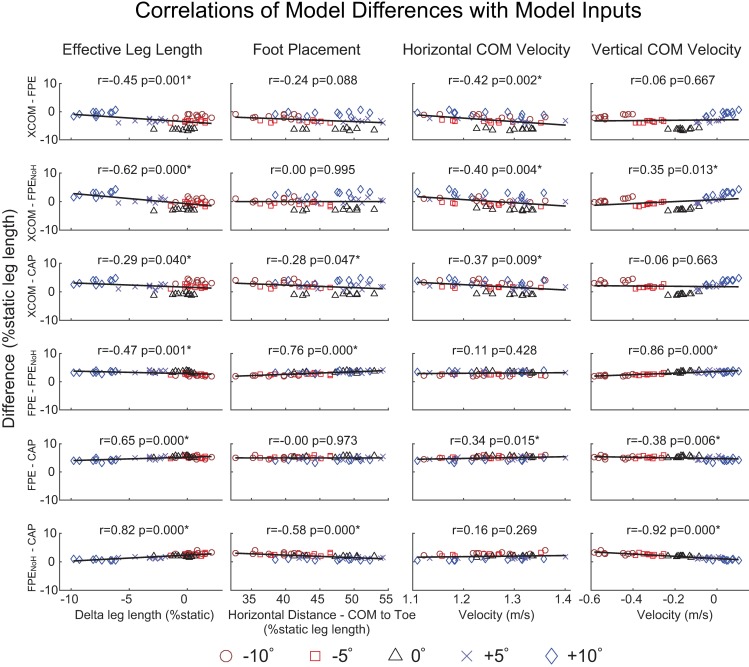
Correlation between differences in stability metrics and model inputs. Correlation between the signed differences between each pair of stability metrics and various inputs that affect model predictions. Effective leg length is the distance from the body center of mass (COM) to the ankle joint center at the instant of heel strike. Foot placement refers to the horizontal (i.e., anteroposterior) distance between the body COM and toe marker at the instant of heel strike. Each stability metric was normalized to percent static (i.e., standing) leg length. Mean values for each subject are plotted for each ramp angle: -10° (red circles), -5° (red squares), 0° (black triangles), +5° (blue crosses), and +10° (blue diamonds). Pearson correlation coefficients (r) and *p*-values are given for each subfigure. Statistically significant correlations are indicated by *.

Effective leg length at heel strike was significantly correlated with all differences between metrics (p≤0.04, Fig 5). There was a strong correlation with FPE_NoH_-CAP (r = 0.82, p<0.001), with larger differences being associated with smaller changes in effective leg length relative to static. There were moderate correlations between effective leg length and XCOM-FPE_NoH_ (r = -0.62, p<0.001) as well as FPE-CAP (r = 0.65, p<0.001). Horizontal COM velocity at heel strike was significantly correlated with several differences, but the correlations were weak (|r|≤0.42, p≤0.015). Vertical COM velocity at heel strike was strongly correlated with FPE-FPE_NoH_ (r = 0.86, p<0.001) and FPE_NoH_-CAP (r = -0.92, p<0.001). Foot placement was moderately correlated with FPE-FPE_NoH_ (r = 0.76, p<0.001) and FPE_NoH_ (r = -0.58, p<0.001).

## Discussion

In this study we used experimental data from ten able-bodied individuals walking on various slopes to investigate differences between stability metrics based on four different formulations of an inverted pendulum model (XCOM, FPE, FPE_NoH_, CAP). We hypothesized that there would differences between metrics on slopes but not on level ground, that differences between metrics would be greater at the extreme slopes (±10°), and that changes in effective leg length would be significantly correlated with differences between metrics.

### Stability metrics based on different models are significantly different on nearly all slopes

We found that, in general, all the model formulations produced stability metrics that were statistically different at each slope angle studied. In general, CAP produced the most negative stability metric values while FPE produced the most positive values (Fig 2). These findings regarding the relationship of the various stability metrics indicate that CAP is the most conservative model formulation (i.e., most likely to indicate an “unstable” step) while FPE is the least conservative (i.e., most likely to indicate a “stable” step). These findings are consistent with prior analyses of three-dimensional versions of the FPE and CAP on level ground, which suggested that the true stability of a human likely lies within a region bounded by the FPE and CAP [27]. The model used to calculate FPE is the only formulation that includes angular momentum, and removing the angular momentum term (FPE_NoH_) resulted in more conservative (i.e., more negative) stability metric values. Angular momentum is an important quantity in the regulation of balance [28,29], and our findings suggest that the presence of angular momentum may help to increase stability. However, in the model used to calculate FPE the angular momentum term is based on the whole-body angular momentum of the person at the instant of heel strike. Humans are capable of actively generating and controlling angular momentum to regulate balance throughout movement [28]. Certain derivations of inverted pendulum models incorporate a flywheel that represents the ability to actively generate momentum [17], which may be used in future research to more accurately represent the ability of a person to come to a stop at any given point in the gait cycle.

### Largest differences did not necessarily occur at extreme slopes

We had expected that the differences between metrics would be smallest on level ground, where the human body behaves most similarly to an inverted pendulum. Contrary to our hypothesis, the overall greatest magnitude of differences between models did not occur at the extreme slopes tested, but on level ground in XCOM-FPE (Fig 4). The mean static leg length for the participants was 0.97 (SD = 0.05) m, thus the results expressed as a percentage of static leg length can be interpreted as roughly equivalent to centimeters. Qualitatively, we did observe the expected V-shaped trend in the differences between XCOM and the other metrics (Fig 3), although the smallest magnitude of differences did not occur at the 0° slope. We also found that the XCOM was more negative (more conservative) compared to the other three metrics on level ground, but as the magnitude of slope angle increased the XCOM tended to become more positive (less conservative) relative to the other metrics. This behavior of XCOM relative to other metrics suggests that there are systematic changes in the outputs of the underlying inverted pendulum models caused by ramp angle.

### No clear overall relationship between models inputs and stability metrics

Despite finding significant correlations between effective leg length and the metric differences, we did not identify a clear relationship across all inverted pendulum-based metrics. Only one of the correlations with effective leg length was considered strong (r = 0.82, FPE_NoH_-CAP). In addition, the greatest changes in effective leg length occurred at -10°, but this condition was not necessarily associated with the greatest differences between metrics calculated using different model formulations (Fig 5).

The strongest overall correlation coefficients were found for the vertical COM velocity at heel strike (r = -0.92, FPE_NoH_-CAP), which may be related to differences in representation of COM motion. The method used to calculate FPE_NoH_ assumes a collision in which angular momentum is conserved, while the calculation of CAP, in contrast, does not model foot-ground collision but forces the COM to follow the experimentally measured trajectory. There was also a strong correlation of FPE-FPE_NoH_ with vertical COM velocity at heel strike (r = 0.86), suggesting that the angular momentum associated with downward (negative) vertical velocity at heel strike plays a role in the differences between these two models. Interestingly, the correlation with vertical COM velocity was much weaker in FPE-CAP (r = -0.38, p = 0.006), despite the fact that CAP neglects angular momentum, similar to FPE_NoH_. Furthermore, the magnitude of FPE-CAP was relatively consistent across all slopes (Fig 4), and was weakly correlated (|r|≤0.38) with all quantities investigated except effective leg length (r = 0.65). These findings regarding FPE-CAP may further support the idea that the “true” stability metric generally lies within the bounds of the FPE and CAP [27]. Also, though our linear correlation analysis returned non-significant or weak correlations for the XCOM comparisons to other metrics, a nonlinear relationship may exist between XCOM and the other metrics as a function of vertical COM velocity. This potential nonlinear relationship may be due to differences in how each underlying model represents the COM trajectory. The model used to calculate XCOM effectively constrains the COM to move along a circular path about the ankle joint, thus neglecting any downward velocity of the COM at the moment of heel strike. The model we used to calculate FPE more appropriately applies conservation of momentum at heel strike, thus accounting for downward COM velocity while still imposing a circular trajectory. The model we used to calculate CAP is likely the most realistic, forcing the COM to follow its experimentally-derived trajectory. However, there are even more model features that could be included. For example, other studies have calculated CAP using a model that includes a flywheel to model torso motion [17] and foot-ground collision can be modeled using alternative formulations to the one we used to calculate FPE [30], which would further affect the calculated stability metrics. Furthermore, passive inverted pendulum models can be modified to incorporate active force-producing components (e.g., [31,32]) that may be more appropriate for assessing ramp walking, though selecting actuator parameters to accurately model specific individuals may be difficult. Further work is needed in order to gain a more comprehensive understanding of the relationship between model characteristics and changes in stability metrics.

Another consideration is that while we compared stability metrics based on various inverted pendulum models to each other, it remains unclear what should be the “gold standard”, or true stability metric. One method for validating inverted pendulum models for assessing human walking on ramps would be to compare human foot placement during gait termination (i.e., stopping) to inverted pendulum model predictions of where the foot should be placed in order to bring the body COM to rest. Studies of gait termination have been performed on level ground [16], but have not yet been applied to ramp walking. In addition to experimental validation, a sensitivity analysis using simulated data could lead to further insights regarding the effect of various model inputs (e.g., COM velocity, effective leg length) on stability metrics. Another approach to assessing the validity of inverted pendulum models could be to analyze correlations between the stability metrics and other balance assessments, as Vistamehr et al. have done for individuals post-stroke [33]. Methods of assessing balance such as whole-body angular momentum [28,29] rely on less simplified representations of the human body and may provide a more complete description of balance on slopes. Thus, while our study examined differences between stability metrics based on inverted pendulum models of human ramp walking, we advise caution when using these models to evaluate human walking on non-level surfaces until further validation is performed.

## Conclusions

We compared stability metrics based on various inverted pendulum model formulations to each other on different slope angles. Contrary to our hypothesis, we found the greatest magnitude of differences between models (XCOM-FPE) on level ground, not on the extreme slope angles. Relatedly, the inclusion of angular momentum in the underlying inverted pendulum model had a greater effect on stability metrics than violations of effective leg length assumptions. Clinical providers and researchers should therefore be aware that inverted pendulum models such as those used to compute XCOM (i.e., margin of stability) and CAP, which neglect angular momentum, are likely to underestimate a person’s sagittal-plane stability, even on level ground. In addition, while we did not identify a clear overall relationship between inverted pendulum model inputs and stability metrics, the vertical velocity of the body COM was correlated with metric differences. Overall, our results suggest that the “true” stability metric value is likely bounded by the FPE and CAP, consistent with prior studies of level-ground walking. However, future work should focus on validation studies (e.g., model predictions of gait termination, sensitivity analyses, correlation with other balance measures) to gain a more comprehensive understanding of inverted pendulum models of human walking on slopes.
